# Spontaneous Fragmentation of Urinary Calculi

**Published:** 1907-11-30

**Authors:** 


### SPONTANEOUS FRAGMENTATION OF URINARY CALCULI.

There can be little doubt that urinary calculi which are giving rise to acute troubles, such as repeated attacks of severe colic, hæmaturia, pyuria, or sleeplessness, should in practically every case be treated by operation—lithotrity, lithotomy, or nephrotomy, as the case may be. There are, however, many cases in which the patient refuses operation, and many others in which both patient and medical adviser would be only too glad if relief could be brought about by non-operative measures.

It is certain that dieting, and the imbibition of more than the usual quantities of spa waters at places like Carlsbad, have a very beneficial effect in calculous cases. One has a sort of feeling that urinary calculi must surely become smaller and smaller, and presently be passed, if only the urine can be kept dilute enough and in such a condition as to contain a minimum percentage of both inorganic and organic constituents.

One also feels that, provided a patient will honestly restrict his proteid diet, and will conscientiously drink abundantly of good plain water containing a minimum of salts, particularly salts of calcium, there is really no reason why the benefits of the treatment should not be as great at home as at Carlsbad. There would then be no reason for his daily work to be interrupted, and the course could be continued for a long time instead of for the comparatively short period that an ordinary visit to Carlsbad allows.

### Illustrative Case.

It is, therefore, with the greatest interest that one reads of a case such as that recorded by Dr. T. R. Bradshaw in the *Liverpool Medico-chirurgical Journal*. The patient was 80 years of age. He had led a professional life until he was nearly 70; he then retired, but still lived an active life. When he was 60 years old he began to pass small uric acid calculi per urethram, and this trouble had continued at intervals ever since. The calculi passed in this way were of the "mustard-seed" type. The urine was almost always acid, pale, but otherwise normal, except that on standing it usually gave rise to a cayenne pepper deposit of uric acid crystals. For several years past the bladder had been irritable, with the result that there was frequent micturition at night.

Hæmaturia was noticed for the first time a little over three years ago, and it recurred periodically. The quantity of blood passed was generally small. It was increased by exercise, and it was uniformly mixed with the urine. Everything pointed to there being a renal calculus. This was confirmed by skiagraphy. The $x$-rays showed two calculi in the right kidney. Shortly after this there were some attacks of acute renal colic. Operation was suggested, but it was not thought to be advisable for several reasons, amongst which was the patient's age.

Six months after the renal colic occurred, fragments of calculus began to be passed repeatedly, and this continued for some months. The fragments were not small calculi; they were definitely portions of large calculi. They were concave on one surface, convex upon the other, like bits of a hazel nut that has been crushed to pieces in the nut-crackers. It was clear that the renal calculus was shedding concentric layers from its surface; in other words, it was undergoing spontaneous concentric fragmentation. The patient soon found himself quite restored to health. He could take active exercise without its inducing hæmaturia as it did before. He felt that he had got rid of something. This fragmentation of a renal calculus spontaneously must be a comparatively rare thing; but even the possibility of its occurrence is not to be gathered from the ordinary text-books.

If the stone can break up spontaneously, it must be from disintegration or solution of the material which unites one layer of the stone to another. This being so, it is a welcome confirmation of the belief that calculi may be cured by medicinal and dietetic means.

If the calculi are causing dangerous symptoms, operation should not be postponed, of course; but if the symptoms are not those of danger, and particularly when the patient first comes under observation, it is well worth while to try and cure them by a conscientious course of treatment by dieting and water-drinking, not at a spa, but at home. Dr. Bradshaw's is not the only case of spontaneous fragmentation of urinary calculi upon record; a considerable number have been collected together from the literature in Dr. Bradshaw's paper.

### Treatment.

The treatment adopted in his case was very simple. The calculi were composed almost entirely of uric acid. The objects aimed at were, therefore, to reduce the nitrogenous intake to the smallest amount compatible with the maintenance of nutrition, to keep up a free flow of urine, and to maintain the general health by moderate exercise, regularity of life, and the avoidance of fatigue. He gave up drinking wine, ate vegetables, white fish, and milk puddings to his lunch and dinner, and only partook of butcher's meat occasionally. Potassium citrate was given to the extent of 5 grains once or twice a day from time to time, especially when pieces of calculus were being passed; he never had enough to make the urine alkaline.

Healthy urine is so constituted that it is seldom in a state of complete saturation even when cold. At the temperature of the body it is probably one of the most powerful solvents that we can hope to possess for all the ingredients that are likely to exist in urinary calculi. The circumstances which Dr. Bradshaw regards as having combined to bring about the disintegration of the calculi in his case, and in others like it, are very simple, but probably they are not frequently found together. They are:—

1. The maintenance of the urine in a healthy condition;
2. The presence in the urine of abundance of water; and
3. The reduction to a minimum of the products of nitrogenous waste.

